# Short-Term Effects of Low-Fat Chocolate Milk on Delayed Onset Muscle Soreness and Performance in Players on a Women’s University Badminton Team

**DOI:** 10.3390/ijerph19063677

**Published:** 2022-03-19

**Authors:** Maryam Molaeikhaletabadi, Reza Bagheri, Mohammad Hemmatinafar, Javad Nemati, Alexei Wong, Michael Nordvall, Maryam Namazifard, Katsuhiko Suzuki

**Affiliations:** 1 Department of Sport Science, Faculty of Education and Psychology, Shiraz University, Shiraz 1585-71345, Iran; m.molaei392@yahoo.com (M.M.); nemati_phy@yahoo.com (J.N.); 2 Department of Exercise Physiology, University of Isfahan, Isfahan 81746-73441, Iran; will.fivb@yahoo.com; 3 Department Health & Human Performance, Marymount University, Arlington, VA 22207, USA; awong@marymount.edu (A.W.); mnordval@marymount.edu (M.N.); 4 Department of Health and Sports Medicine, University of Tehran, Tehran 11155-4563, Iran; maryam_namazifard@yahoo.com; 5 Faculty of Sport Sciences, Waseda University, Tokorozawa 359-1192, Japan

**Keywords:** carbohydrate–protein beverage, chocolate milk, post-exercise recovery, aerobic performance, anaerobic performance, DOMS, badminton players

## Abstract

This study investigated the short-term effects of low-fat chocolate milk (LFCM) consumption on delayed onset muscle soreness (DOMS) and performance in female badminton players. Seven female badminton players (23 ± 1 years; height: 163.8 ± 4.1 cm; body mass: 58.7 ± 0.9 kg) were randomly assigned to 1 week of LFCM (500 mL) or placebo (water, 500 mL) consumption in a crossover design. Participants consumed LFCM or water immediately after each training session during the 1-week intervention. Performance variables (aerobic power, anaerobic power, agility, explosive power, and maximum handgrip strength) were assessed at two separate time points: pre and post-intervention (after 1 week). In addition, the Visual Analogue Scale (VAS) was used to assess DOMS before, immediately after, and at 24 and 48 h after each training session. There were significant time effects for aerobic power, upper body explosive power, minimum anaerobic power, and time to exhaustion (TTE), which significantly increased after LFCM consumption (*p* < 0.05). Moreover, relative and maximum lower body power significantly (*p* < 0.05) increased, while rating of perceived exertion (RPE) as well as DOMS in lower extremity muscles immediately after exercise significantly decreased after LFCM consumption compared to placebo (*p* < 0.05). There were no significant changes in maximum anaerobic power, agility, and maximum handgrip strength (*p* > 0.05). LFCM, as a post-exercise beverage, may help speed recovery in female badminton players leading to increased aerobic, anaerobic, and strength performance indices, increased TTE, and decreased muscle soreness and RPE.

## 1. Introduction

Badminton is a high-intensity racket sport that imposes considerable metabolic and mechanical loads on bodily systems [1,2]. High-level performance in badminton requires speed, power, agility, flexibility, endurance, and strength, measurably involving both aerobic and anaerobic energy systems in intense, short- and long-term exercise [1,2,3]. Single-player matches tend to be significantly more demanding than doubles matches, with 80% of exercise efforts lasting less than 10 s [1,2,4,5,6,7,8]. Badminton is considered a safe and contactless sport involving rapid direction changes, jumps, lunges toward the net, and rapid arm movements to strike the shuttlecock in various positions [9]. Moreover, badminton-induced delayed onset muscle soreness (DOMS) often significantly affects athletes’ performance and recovery.

DOMS is described as delayed muscular pain resulting from muscular structure injury after training sessions or strenuous exercise, especially in eccentric contractions such as badminton [10,11]. Clinical symptoms and signs of DOMS include myalgia, tenderness, and decreased articular range of motion leading to unavoidable adverse effects on an athlete’s performance [10]. Signs and symptoms of DOMS increase progressively after exercise, peaking at 24–72 h post-exercise and gradually disappearing 5–7 days post-exercise. Although DOMS is not a serious problem, the associated discomfort can reduce participation in training sessions and/or sports performance [10]. Strategies to aid recovery when experiencing DOMS should be considered to reduce the risk of more severe injury during higher training loads or return to athletic competition following tapering and/or off-season [10]. Therefore, several modalities have been proposed as having the potential to alleviate symptoms of DOMS and accelerate muscle recovery in order to optimize athletic performance and fitness in a variety of sports, including badminton [10,11,12].

Post-exercise dietary protein ingestion has been recommended to maximize muscular performance and body composition [13,14,15,16]. For instance, chocolate milk has been touted as an effective and accessible performance and recovery aid due to its mass availability, cost-effectiveness, and similar caloric content and carbohydrate–protein ratio as many commercially available (and more costly) recovery beverages [17]. For example, Karp et al. showed that low-fat chocolate milk (LFCM) consumption could increase time to exhaustion (TTE) during cycling by approximately 15 min compared to a commercially available recovery aid [17]. Investigations have also reported that the protein content in chocolate milk may reduce the symptoms of exercise-induced muscular injury [18,19,20] and attenuate DOMS [21,22,23]. However, perhaps due to study design differences, other investigations were not as clear on the effect of chocolate milk consumption on DOMS [18,19,20]. Ultimately, for athletes with limited recovery time (8 h or less), co-ingestion of carbohydrate and protein in the form of chocolate milk may have a significant influence on restoring glycogen stores through insulin-mediated pathways as well as promoting post-exercise muscle reconditioning due to its protein and amino acid content [24].

The ergogenic effects of chocolate milk on athletic performances requiring strength, anaerobic efforts (power), and post-exercise recovery have received less attention. Utilizing a crossover study design, Potter et al. compared the effects of chocolate milk and water as recovery aids after a debilitating bout of high-intensity endurance climbing on 10 male rock climbers, finding that chocolate milk consumption reduced muscle soreness and increased indicators of performance [25]. Yet, there is limited evidence regarding the effects of chocolate milk consumption on performance and recovery in other athletic pursuits such as badminton. A study investigating the effect of low-fat lactose-free milk on badminton performance showed that free consumption of low-fat lactose-free milk, a carbohydrate–electrolyte sports drink, or water while exercising had relatively similar effects on hydration status, performance, and other physiological variables. It should be noted that participants in this study gravitated towards their preferred drink, in this case, the sports drink, which resulted in greater ad libitum consumption [26]. Moreover, while an important consideration, low-fat lactose-free milk consumption in this study during exercise caused thirst and gastrointestinal discomfort compared to the sports drinks and water but had no negative effects on hydration, performance, and recovery [26]. Strategically, it seems that ad libitum consumption of milk in particular and other sports drinks should be monitored or further investigated to reduce or eliminate unwanted side effects in athletes. One suggestion has been to limit milk consumption to 500 mL. In doing so, after repetitive speed exercises that induced muscular injury, declines in dynamic muscle function were lessened, thus accelerating recovery and performance in subsequent bouts of exercise despite there being no perceived difference in DOMS post-exercise [27]. Nutritional interventions improve muscle injury treatment, increase replenishment of endogenous fuel stores such as muscle and liver glycogen, and include high carbohydrate availability (chocolate milk contains upwards of 60 g of carbohydrate per 500 mL). During post-exercise recovery, these interventions have become common practice for endurance athletes in particular. Short-term recovery (8 h or less) appears to require 1.2–1.5 g/kg/h of carbohydrate intake to maximize glycogen synthesis after exercise. Further, rapid (e.g., within 30 min post-exercise) carbohydrate ingestion at regular intervals (e.g., every 15–30 min) has shown to maximize muscle glycogen synthesis by maintaining high plasma glucose and insulin concentrations. As such, delaying carbohydrate ingestion by 2 h or more at the conclusion of exercise lowers glycogen levels and delays recovery [24].

Given the importance of applying an effective nutritional strategy to enhance recovery and considering the ambiguous results on the effectiveness of chocolate milk, the present study aimed to investigate the effects of LFCM consumption on performance, recovery, and DOMS in badminton players. 

## 2. Materials and Methods

### 2.1. Participants 

Seven female students (age: 23 ± 1 years; height: 163.8 ± 4.1 cm; body mass: 58.7 ± 0.9 kg; body mass index (BMI): 21.9 ± 3.5 kg/m^2^) who played on the women’s badminton team at the Shiraz University of Medical Sciences volunteered to participate in this study. The participants self-reported via health and exercise history questionnaires having at least 5 years of experience playing badminton, no history of allergy to LFCM, sleeping at least 7–8 h during the 24-h day, and not taking any supplements or medications. Moreover, the participants were reportedly non-smoking and did not consume alcoholic or caffeine-containing beverages at the time of data collection. All participants gave written informed consent before their inclusion in the research. This study was reviewed and approved by the Ethics Committee of the Faculty of Rehabilitation Sciences, Shiraz University of Medical Sciences, Shiraz, Iran (ethics approval code: IR.SUMS.REHAB.REC.1399.041) and carried out in accordance with the Declaration of Helsinki.

### 2.2. Study Design

**Figure 1:**
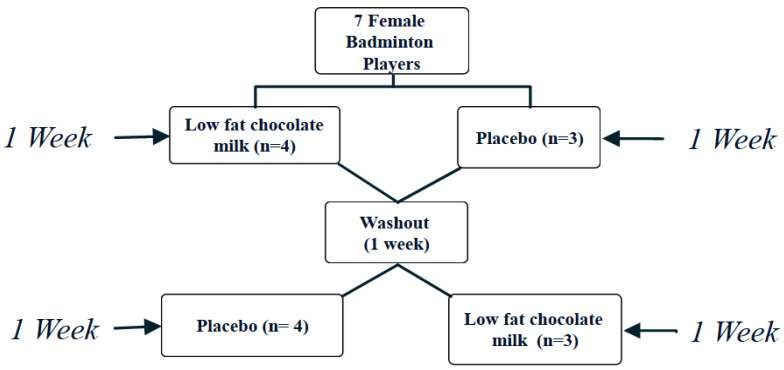
Schematic study design.

The present study was a randomized, two-period, crossover design (Figure 1). On the first visit, the participants obtained familiarization with the tests (SEMO Test, Medicine ball throw test, Sargent jump, Grip strength, RAST, and the YOYO intermittent test) following the initial screening process. Testing was conducted in the morning in a quiet temperature-controlled room (22–24 °C) after avoiding caffeinated drinks and alcohol for at least 24 h. The tests were completed at the same time of the day (7–9 h) for each participant to diminish possible diurnal variations in the examined parameters at baseline and the end of each treatment. Participants were also instructed to avoid extraneous intense exercise for 2 days before and after each trial to control for any acute and ancillary effects of exercise on the measured variables [28]. Following baseline measurements, participants were randomly assigned to drink 500 mL water (placebo) or 500 mL of LFCM after each training session for one week, separated by one week washout period in which participants continued their training regimens. The LFCM consisted of fresh cow’s milk, sugar, and cocoa powder; its nutritional values are presented in Table 1. The dose of LFCM of ~500 mL has shown to be efficacious to increase TTE [17,29,30] and decrease muscle soreness [28]. Participants abstained from consuming drinks containing milk and/or caffeine for the duration of the study [28]. Water was selected as the placebo to aid in fluid loss associated with training sessions and a lack of suitable substitute for chocolate milk, as discussed in prior literature [25,28,31,32]. 

### 2.3. Anthropometrics

Height and body mass of all participants were measured using a stadiometer (Seca, Hamburg, Germany) and a scale (Beurer, Germany; model: PS06) with a reported accuracy of 0.1 cm and 0.01 g, respectively. Body mass index (BMI) was calculated using the Ancel Keys equation by dividing body mass (kg) by height squared (m^2^). 

### 2.4. Performance Indicators 

The YOYO intermittent test was used to assess the aerobic power and TTE [33], while anaerobic power was measured using the Running Anaerobic Sprint Test (RAST) [34]. The Southeast Missouri (SEMO) test [35] was used for agility assessment. The 3-kg medicine ball throw test [36] and vertical jump test (Sargent jump) [33] were used to assess the explosive power of upper and lower extremity muscles, respectively. The relative (e.g., adjusted for body mass) and maximal power of the lower extremity were evaluated using the Johnson and Bahamonde formula [33], while a handgrip dynamometer (Jamar Hydraulic Hand Dynamometer, Warrenville, IL, USA) was used to measure the maximal isometric grip strength [33,37,38]. Except for the YOYO and RAST tests, all assessments were performed three times consecutively, with the best score recorded as the final result. The tests were performed following the National Strength and Conditioning Association [39] in the following order: SEMO Test, Medicine ball throw test, Sargent jump, Grip strength, RAST, and the YOYO intermittent test. All performance tests were completed before beginning the protocol (baseline) and at the end of the 1-week intervention (LFCM or placebo).

### 2.5. Delay Onset Muscle Soreness

The Borg Rating of Perceived Exertion (RPE) scale [40] and the Visual Analogue Scale (VAS) [41] were used to assess the DOMS of the lower and upper extremity muscles. VAS questionnaires of the upper and lower extremity muscles were completed prior to (just before the exercise session), immediately after, and then at 24 and 48 h after each training session. RPE was measured just immediately after each session. 

### 2.6. Training and Intervention Protocol

Participants underwent a 3-week (includes the washout period) training protocol consisting of 4 times per week of three 90-min sessions of badminton training (play, drills, and strategy) and a 60-min conditioning session designed for badminton players (speed, agility, and plyometric exercises). The time and location of the training were identical for all participants and were under the supervision of a certified trainer to ensure safety and adherence to the training protocol. 

### 2.7. Statistical Analyses

All data were analyzed using descriptive and inferential statistical methods. The data distribution normality was determined using the Shapiro–Wilk test. Differences between interventions and baseline data were performed using crossover study ANOVA utilizing SPSS software (v. 24.0, IBM; Chicago, IL, USA). Statistical significance level was considered at *p* ≤ 0.05.

## 3. Results

**Figure 2:**
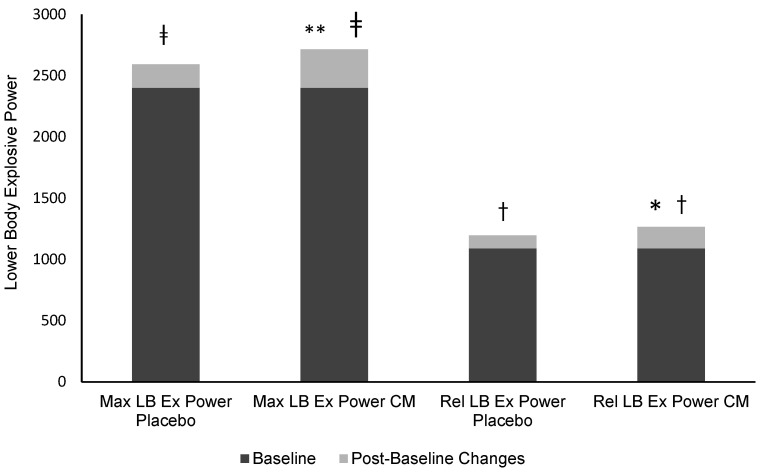
Subjective measurements of maximum and relative explosive power of lower extremity muscles on post-test–placebo and post-test–LFCM. Values are mean (* = Significantly different (*p* < 0.05) from maximum explosive power week placebo, ** = Significantly different (*p* < 0.05) from relative explosive power week placebo, † = Significantly different (*p* < 0.05) from relative explosive power baseline, ‡ = Significantly different (*p* < 0.05) from maximum explosive power baseline).

**Figure 3:**
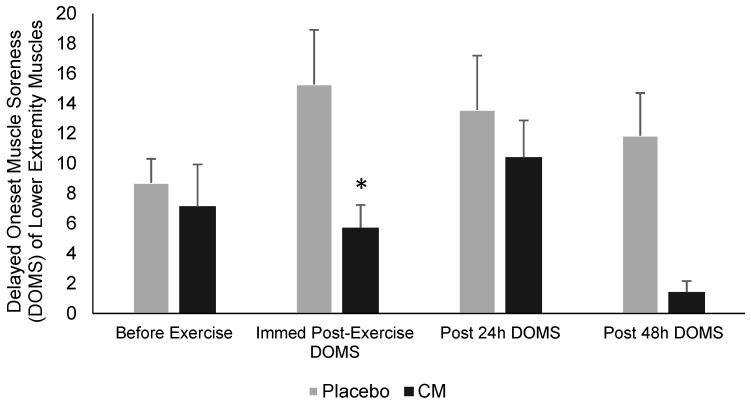
Subjective measurements of delayed onset soreness of lower extremity muscles before exercise, immediately post-exercise, 24 h post-exercise, and 48 h post-exercise for each training session of week placebo and week chocolate milk. Values are mean ± SD (*p* < 0.05) (* = Significantly different (*p* < 0.05) from week placebo).

Characteristics of the seven female students playing on a women’s university badminton team included in this study (average (± SD) body mass and BMI were 58.7 ± 0.9 kg and 21.9 ± 3.5 kg/m^2^, respectively) are presented in Table 2. All the performance variables at baseline and after a week of LFCM consumption or placebo are shown in Table 3. There were significant time effects for aerobic power, upper body explosive power, minimum anaerobic power, and TTE, which significantly increased after LFCM consumption (*p* < 0.05). Moreover, relative and maximum lower body power (Figure 2) significantly increased (*p* < 0.05) after LFCM consumption compared to placebo. There were no significant changes in maximum anaerobic power, agility, and maximum handgrip strength (*p* > 0.05). Regarding DOMS, participants had less muscle soreness at 24 and 48 h after exercise in the LFCM intervention (*p* > 0.05). Moreover, following the LFCM there was significantly less lower extremity muscle soreness immediately after exercise (*p* < 0.05; Figure 3) in the subsequent days, which could promote a more rapid return to training and performance. Furthermore, there was a significant decrease in RPE compared to placebo (*p* < 0.05) (Table 4).

## 4. Discussion

The present study intended to investigate the short-term effect of LFCM consumption on DOMS and performance of female students playing university level badminton. Briefly, our major findings were as follows: (1) maximum, relative, and lower body power significantly increased, while RPE as well as DOMS in lower extremity muscles immediately after exercise significantly decreased after LFCM consumption compared to placebo; (2) there were also improvements in aerobic power, upper body explosive power, minimum anaerobic power, and TTE significantly after LFCM compared to baseline.

It has been reported that post-exercise nutrition is essential for hepatic and muscular glycogen store replacements, restoration of fluids and electrolytes lost during exercise, injured muscular tissue repair, muscle protein synthesis (MPS) stimulation, and musculoskeletal adaptation [22]. Badminton athletes are accustomed to several training sessions daily, with about 6 h of recovery time between sessions, and thus complete and rapid recovery is critical to optimize subsequent performances [42,43,44]. LFCM contains 90% water and a carbohydrate-to-protein ratio of 4:1. As a recovery-supporting drink, chocolate milk aids in glycogen restoration, MPS, and rehydration and may significantly reduce lactate accumulation during exercise [31,45]. Central fatigue is likely diminished by carbohydrate and protein drinks (CHO + PRO) because decreased glucose leads to tryptophan release, increasing the cerebral serotonin and subsequently triggering fatigue in the central nervous system [22,46]. In fact, the fat contained in LFCM has been shown to increase circulating fatty acid levels, thus acting as glycogen sparing fuel during exercise [20,22,47]. The present study found that LFCM increased TTE in the YOYO intermittent test by 24% compared to the placebo intervention. Such short-term improvements in aerobic power are more likely the result of dietary supplementation as discussed above (e.g., increased glucose and free fatty acid availability, lessened exercise-induced muscle damage, etc.) than the often-cited stimuli from prolonged exercise training [17,30,48]. As noted in Table 1, the fat content is higher in chocolate milk than typical carbohydrate-based energy recovery drinks, which frequently contain upwards of 2.7g of fat, perhaps leading to the significant observed effects on TTE in the present study [22,49]. Further, short-term increases in maximum oxygen consumption (VO_2max_) may result from acute cardiovascular alterations and oxygen exchange rate, which in turn, can improve skeletal muscle function via oxygen transfer and utilization [48,50,51].

Timing and composition of pre-exercise nutrition are equally paramount to aid recovery after intense bouts of exercise [18,52]. As a result, increased carbohydrate consumption immediately after exercise helps in the rapid replacement of muscular glycogen [18,53], which decreases RPE and prevents decrements in athletic competition [18,54]. In the present study, an 11% decrease in RPE was observed after a short period of LFCM consumption compared to water which may follow that protein consumption in combination with carbohydrates has a greater effect on muscle injury repair, recovery facilitation of muscular functions (such as muscle strength, power, and soreness), and MPS [23,55]. Dairy products are rich in amino acids, proteins, fats, minerals, and vitamins, giving them added health and recovery benefits [23,56], likely due to its constituent lactose (carbohydrate), casein, and whey protein (whey-to-casein ratio of 3:1).

Moreover, dairy products such as chocolate milk are a good source of calcium, sodium, and potassium, which support post-exercise fluid (rehydration) and electrolyte restoration [56], both essential for proper muscle function. In addition to increasing physiological strain, exercising in dehydrated conditions can reduce physical and cognitive performance. It is hypothesized that the high nutritional density of chocolate milk promoted a slower gastrointestinal transit, a longer and more evenly distributed water absorption in the intestines, and acute circulatory hypervolemia and consequent avoidance of diuresis. Certainly, each area deserves further attention, and prior research investigating the hydration capacity of carbohydrate–protein drinks following 2% mean body mass loss found improved fluid retention and/or enhanced fluid balance when the recovery beverage was consumed without extra food or fluid [57]. This may help explain the findings in the present study of a 62.6% reduction in early DOMS of lower extremity muscles following the chocolate milk consumption compared to water alone. Subsequent research would do well to investigate hydration status and cardiovascular effects (such as plasma volume and associated albumin levels) following repeated bouts of exercise over time relative to chocolate milk and other nutritionally-based recovery strategies [50,55]. 

Maximum and mean anaerobic power did not change significantly after LFCM consumption in the present study; however, the minimum anaerobic power of participants following repeated bouts of sprinting (RAST) significantly increased by 18.2% compared to the water placebo. As observed in badminton competition, repeated bouts of high-intensity physical activity eventually led to muscle fatigue and subsequent decrements in athletic performance. Several causes of fatigue have been suggested, including the onset of blood lactate and/or other biochemical metabolites, neurological impairments at the musculoskeletal junction, and/or depleted energy (carbohydrate and fats) stores and availability [58,59]. As exercise continues, the body encounters a physiological environment consisting of dehydration, glycogen depletion, low blood insulin concentrations, increased cortisol levels, and other catabolic hormones [59,60]. As a recovery strategy, introducing milk-based beverages including LFCM, which is rich in nutrients needed for maintaining bodily energy reserves, may enhance protein, carbohydrate, fluid, and vitamin/mineral availability, thus lessening fatigue during repeated or subsequent bouts of exercise [59,61]. Future research should address longer-term LFCM consumption compared to other nutritional strategies on the preservation of and/or improvements in maximum anaerobic power following repeated exercise; 1 week may not have been sufficient to observe such differences with the present study design.

The protein content of milk and LFCM facilitates amino acid availability in muscles, increasing MPS [28,62]. Even in the short-term post-exercise, MPS is upregulated, as observed by decreased muscle protein breakdown markers and activation of signaling pathways [28,29,63]. For example, in recovery, 0.4 g/kg of chocolate milk causes a higher insulinemic impact and increases activation of MPS pathways as observed by increased mechanistic target of rapamycin (mTOR) phosphorylation. Further, as a source of high-quality protein, given its leucine and other essential amino acids (EAAs) content in combination with carbohydrates [64,65], chocolate milk may positively impact insulin response and MPS signaling pathways in skeletal muscle. As such, nutrient- and contraction-induced activation of insulin-like growth factor (IGF) and other MPS signaling pathways after endurance, resistance, and concurrent training protocols, increases with additional post-exercise protein consumption [57]. 

Given the nutritional effect of milk on MPS and the presence of EAAs and fat (milk fat has been suggested to increase the absorption of fat-soluble vitamins A, D, and E), maintenance or even gains in strength upon subsequent bouts of exercise are not uncommon in the literature [64,65]. Such reasoning may help explain the 16% increase in the explosive strength of upper extremity muscles and the 16.2% and 13.1% relative and maximum explosive power increases of upper extremity muscles, respectively, compared to water alone. Furthermore, while not statistically significant, it is worth noting that maximum grip strength, an important performance indicator in racket sports, including badminton, increased 12% compared to placebo. Indeed, this and other indices of performance in the present study, such as agility which was unchanged with chocolate milk consumption [59], may have shown more significant alterations compared to the literature [60] when considering the limitation of study participation. 

There are several limitations to this study, such as the lack of an equicaloric placebo. However, water was used as the placebo due in part to aid in fluid loss associated with training sessions and a lack of suitable substitute for chocolate milk as discussed in prior literature [25,28,31,32]. In addition, we did not control post-exercise food consumption. Moreover, we had a small sample size. Yet, it could be argued that most published research in badminton had a low participant number, which may be related to the modest size of most badminton teams [17,30,31,66,67,68]. Furthermore, the amino acid and vitamin composition of the supplemented milk was not provided, which might aid in the interpretation of our results. Another limitation is that although we measured performance markers, actual in-game performance was not assessed, and consequently, future studies should evaluate this component. 

## 5. Conclusions

Our findings are comparable to prior literature on the benefits of LFCM as an effective recovery drink after repeated bouts of high-intensity exercise [17,30]. We found that LFCM had a significant effect on TTE, RPE, and DOMS following a week-long training session compared to placebo. Moreover, consuming LFCM during recovery improved aerobic and anaerobic power measures of female students playing on a university women’s badminton team.

## Figures and Tables

**Table 1:** Energy and nutrient composition of LFCM (500 mL).

Energy (Kcal)	370
Fat (g)	7.5
Protein (g)	16
Carbohydrate (g)	59.5
Calcium (mg)	600
Phosphorus (mg)	500

**Table 2:** Participant characteristics.

Age (y)	23.14 ± 1.5 years
Height (cm)	163.8 ± 4.1 cm
Body mass (kg)	58.7 ± 0.9 kg
Body mass index (kg/m^2^)	21.9 ± 3.5 kg/m^2^

**Table 3:** Subjective measurements of YOYO intermittent test (Time To Exhaustion (TTE), aerobic power (VO_2max_ (mL/min/kg))), RAST test (maximum anaerobic power (Watt), minimum anaerobic power (Watt), average anaerobic power (Watt)), SEMO Test (Agility (sec)), medicine ball throw test (explosive power of the upper body (meter)), handgrip dynamometer (max hand strength (kg)), Vertical Jump Test (relative explosive power of the lower body (Watt), maximum explosive power lower body (Watt)) on baseline, post-test placebo and post-test LFCM. Values are mean ± SD (*p* < 0.05) (* = Significantly different (*p* < 0.05) from baseline, ^#^ = significantly different (*p* < 0.05) from post-test placebo).

		TTE	Aerobic Power	Max Anaerobic Power	Min Anaerobic Power	Ave Anaerobic Power	Agility	Explo Power Upper Body	Max Hand Strength	Rel Explo Power Lower Body	Max Explo Power Lower Body
	
Pre-test (Baseline)		262.8 ± 39	38.6 ± 0.32	266.1 ± 56.4	134.5 ± 28.4	195 ± 43.9	14.8 ± 1.1	4.4 ± 0.5	22.8 ± 4.7	1089.6 ± 258.6	2402.1 ± 508.6
Post-test Placebo		301.4 ±38.4	38.9 ± 0.32	234.1 ± 37.4	141.4 ± 32.6	184.01± 30.3	14.3 ± 0.35	5 ± 0.36	24 ± 4.8	1196 ± 264.7 *	2593 ± 514 *
Post-test LFCM		325.7 ± 67 *	39.1 ± 0.56 *	252.5 ± 64.6	159 ± 35.5 *	200.1 ± 41.1	14.2 ± 0.38	5.1 ± 0.31 *	25.5 ± 3.9	1265.7 ± 304.6 *^#^	2716.5 ± 583.8 *^#^

**Table 4:** Subjective measurements of RPE and delayed onset soreness of upper body and lower body in the placebo and chocolate milk interventions. Values are mean ± SD (*p* < 0.05) (* = Significantly different (*p* < 0.05) from week placebo).

		RPE	DOMS (Upper Body)				DOMS (Lower Body)			
			Before Exercise	IP	Post 24 h	Post 48 h	Before Exercise	IP	Post 24 h	Post 48 h
Week Placebo		11.8 ± 0.69	3.5 ± 0.77	2.18 ± 0.75	0	0	8.71 ± 1.6	15.28 ± 3.63	13.57 ± 3.62	11.85 ± 2.85
Week LFCM		10.5 ± 0.78 *	0	6.42 ± 1.8	0	0	7.14 ± 2.8	5.71 ± 1.52 *	10.42 ± 2.45	1.42 ± 0.74

IP: Immediate post-exercise.

## Data Availability

Data are available upon email to corresponding authors. The data are not publicly available due to faculty ethic roles.

## Practical Applications

LFCM is a healthy and high-quality drink that may have positive effects on recovery after high-intensity intermittent exercise. The post-exercise consumption of LFCM may improve DOMS, RPE, TTE, strength, and aerobic and anaerobic power in badminton players.
